# Bystanders’ willingness to assist using automated external defibrillators during cardiac arrest

**DOI:** 10.1016/j.heliyon.2024.e37316

**Published:** 2024-09-02

**Authors:** Hideko Kono, Koichi Takaishi, Masaya Onuma, Michi Fukushima, Ryosuke Takeuchi

**Affiliations:** a Graduate School of International Social Sciences, Yokohama National University, 79-4 Tokiwadai, Hodogaya-ku, Yokohama-shi, 240-8501, Japan; b Faculty of Business Administration, Asia University, 5-8 Sakai, Musashino-shi, 180-8629, Japan; c Graduate School of Economics, Tohoku University, 27-1 Kawauchi, Aoba-ku, Sendai-shi, 980-8576, Japan

**Keywords:** Out-of-hospital cardiac arrest, Automated external defibrillator, Bystander effect, Bystanders' willingness to rescue, Cardiopulmonary resuscitation

## Abstract

The “bystander effect,” in which the presence of others inhibits rescue actions, has not been specifically examined in the context of cardiac arrest; understanding this effect in relation to rescue with automated external defibrillators (AEDs) is important. This study aims to identify the presence of others as a factor inhibiting rescue actions using an AED, from a social psychology perspective. We collected data through a web-based questionnaire involving registered residents in all 47 prefectures of Japan. The participants were presented with hypothetical scenarios of witnessing cardiac arrest events at train stations, under sparse or crowded conditions, and with or without the presence of competent parties (e.g., station staff or security guards). Their willingness to intervene was assessed across three levels of rescue behavior: (1) running and calling for help, (2) retrieving an AED, and (3) using an AED. This study found evidence of the bystander effect, indicating that the presence of competent others reduced behavioral interventions by bystanders during out-of-hospital cardiac arrest (OHCA) events. Moreover, the perceived presence of competent parties at the scene of a cardiac arrest reduced bystanders’ willingness to initiate rescue under certain circumstances. While many bystanders were willing to initiate rescue efforts in response to calls for help, they resisted rescues involving an AED. This study observes that a bystander effect occurs among bystanders witnessing OHCA, explores the inhibiting effects of identifying competent parties on the initiation of rescue efforts, and suggests that there are significant invisible barriers to using AEDs in rescuing patients with OHCA.

## Highlights

• We investigate the bystander effect on AED use in cardiac arrest situations.
• A web-based survey is used with respondents from Japan for emergency situations.
• Inhibiting effects for identifying competent parties are investigated.
• The presence of competent others reduced intervention during OHCA events.
• A “bystander effect” is observed to occur among bystanders witnessing OHCA.

## 1 Introduction

This study aims to identify the presence of others as a factor inhibiting rescue using an automated external defibrillator (AED) from a social psychology perspective. Social psychology research has introduced the well-known concept of the “bystander effect,” in which the presence of others inhibits rescue actions [[1], [2], [3]]. However, although the bystander effect has not been specifically examined in the context of cardiac arrest, the need for greater knowledge of bystander effects and their effectiveness in mobilizing lay responders to rescue with AEDs has been identified [4,5].

The concept of the bystander effect argues that the presence of others can negatively influence bystanders’ willingness to engage in rescue operations because of factors such as social influence, diffuse responsibility, and pluralistic ignorance [2,3,6,7]. Furthermore, familiarity with the environment [6] and group cohesion [7,8] can facilitate bystander intervention.

Cardiopulmonary resuscitation (CPR) by bystanders is paramount to improving the survival rate of out-of-hospital cardiac arrest (OHCA) patients [[9], [10], [11], [12], [13]]. Increasing bystander use of AEDs to save lives is a critical issue. Previous research has identified a variety of barriers to bystander lifesaving attempts, including psychological barriers such as panic [14], concerns about a lack of CPR knowledge and skills [15], and uncertainty about whether the affected person is experiencing cardiac arrest [16]. Conversely, factors that have been reported to facilitate bystander intervention include prior AED training [[14], [17], [18], [19], [20], [21]] and a high level of CPR competency at the community level [22,23]. Understanding bystander perceptions and motivations can help increase bystander lifesaving rates; however, few existing studies have directly assessed this aspect. In this context, this study focuses on participants’ self-reported expected responses (subjective measures) rather than their actual behavior (objective measures) to analyze their confidence and effectiveness in using an AED.

Recent research suggests that the bystander effect is attenuated in serious emergencies and in situations where bystanders know each other [[24], [25], [26], [27]]. Considering that this study is interested in the mechanism by which the bystander effect occurs, we assumed the scenario of being alone at a train station as a typical situation where the bystander effect is likely to occur. This is because the emergency situation is not life-threatening and there is no psychological relationship between the bystanders. Japan was chosen as the analysis region because it has the second-largest number of AEDs in the world. Although public access to defibrillation has improved survival rates after OHCA [[28], [29], [30]], the proportion of OHCA patients who receive an early shock remains low. Life-saving rates from public access defibrillation also remain low relative to the number of AEDs in place [29,31,32], suggesting the need for the more effective use of AEDs [29,33].

In the selected analysis situation, two hypotheses were formulated for this study. First, studies analyzing the bystander effect have consistently observed that the number of strangers is inversely proportional to the likelihood of occurrence of bystander intervention [[3], [4], [5], [6], [26], [34]].

Hypothesis 1 The greater the number of bystanders, the lower the bystander's willingness to intervene.

Second, the likelihood of intervention in an emergency depends on whether the bystanders are competent parties who play a professional role at the scene of the emergency [35,36]. As bystanders become aware of the presence of others who are competent and willing to intervene, the dispersion of responsibility decreases their willingness to act [37,38].

Hypothesis 2 When bystanders become aware of the presence of competent and involved parties at the location of cardiac arrest, their willingness to initiate rescue activities decreases.

In addition to verifying the above hypotheses, we explore the factors that affect bystander effects of AED usage. Specifically, we examine the relationships between bystanders’ willingness to intervene and their demographic variables, such as gender, age, and AED training experience.

## 2 Methods

We conducted an online survey to evaluate laypersons’ willingness to intervene when witnessing a cardiac arrest in various bystander situations. The survey was conducted using Questant (MACROMILL, INC., Tokyo, Japan), which uses a data pool of registered individuals who have previously agreed to complete surveys. The registered residents in 47 prefectures in Japan were randomly sampled. The survey participants were targeted to uniformly represent men, women, and different age groups.

Although several bystander studies support Utstein's definition, which considers healthcare professionals (doctors, nurses, and paramedics) as being “bystanders” if they are involved in life-saving actions outside their work, we excluded such professionals, as this study focuses on laypersons' rescue behavior.

The study was conducted in accordance with the principles of the Declaration of Helsinki. A detailed information sheet, including the objectives of the study, was provided to the participants. The participants were free to refuse to participate in the study and withdraw from participation. They were informed that the results would be anonymous, and informed consent from the participants was sufficient to conduct the study.

**Fig. 1:**
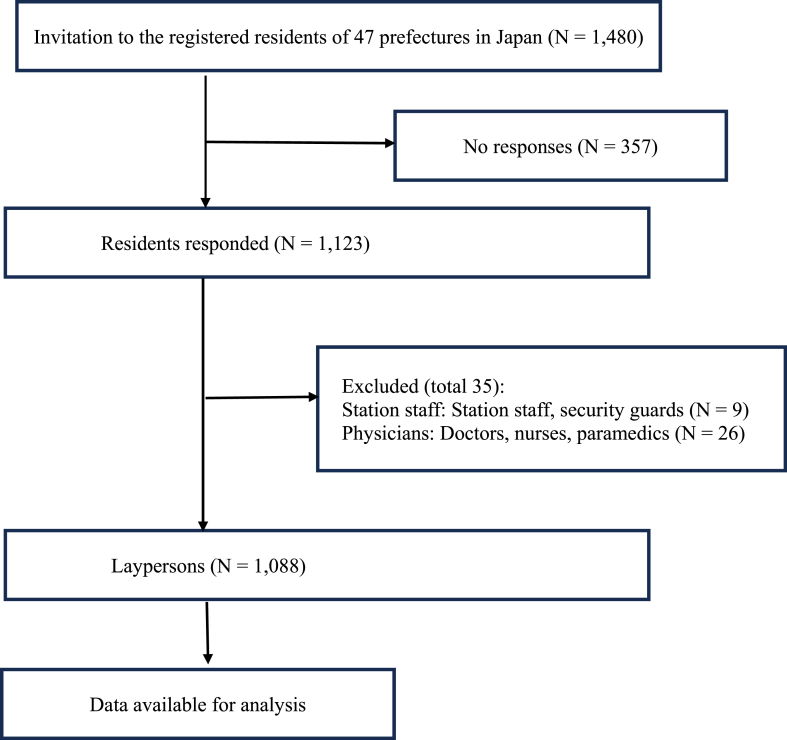
Flow diagram of the survey respondents and their inclusion in data analysis.

Overall, 1480 registered individuals received invitation e-mails containing the link to the questionnaire, along with the study description and purpose, on August 14, 2023. The survey was intended to be terminated when 1000 valid responses had been collected. A total of 1123 responses were obtained from the Internet survey (response rate = 75.8 %). We excluded 35 responses corresponding to station staff and physicians. The final sample for data analysis comprised 1088 responses (Fig. 1).

### 2.1 Questionnaire

We designed a structured questionnaire using information from an extensive literature review and previous interviews with a Japanese AED company, physicians who had rigorously initiated promotions of AED usage among laypersons in Japan, and several chief officers of the fire departments of city municipalities that had actively promoted the use of bystander CPR to reduce OHCA. The questionnaire included questions on demographic data (including age and gender) and whether the individual had received AED training (yes/no and the time of the last training course if the answer was yes).

Subsequently, we presented hypothetical scenarios and asked whether the respondents were willing to intervene when they witnessed a cardiac arrest at a train station to assess both the bystander effect and the effects of competent parties. Respondents were briefed beforehand on cardiac arrest and the effectiveness of AED use. To analyze the bystander effect and compare differences in responses, the questionnaire included both sparse and crowded scenarios. Additionally, the station staff and security guards performed their professional roles as competent parties in the scenarios. We also added kiosk staff as a reference. It was possible to compare any differences in responses, depending on whether bystanders were aware of their presence.

Three levels of rescue behavior were established to thoroughly ascertain attitudes toward respondents’ willingness to (1) rescue by running and calling for help, (2) rescue by retrieving an AED, and (3) rescue by using an AED. For all rescue behaviors, respondents rated their degree of agreement with each item on a 5-point Likert scale ranging from 1 (not willing to) to 5 (willing to). The respondents indicated their willingness to engage in the above-mentioned rescue behaviors under the following scenarios.

(i) Two bystander conditions: sparse (it was a weekday afternoon and there were few people on the platform) or crowded (it was rush hour and the platform was crowded with passengers).
(ii) Three scenarios regarding the presence of persons: station staff and security guards were within visual range; platform kiosk staff were within visual range; and neither station staff and security guards nor platform kiosk staff were within visual range. Participants who answered “willing to” and “probably willing to” were classified as the willing group. The questionnaire was piloted with five researchers, and it was revised as required before the survey commenced.

The sample size for this study was calculated a priori using G*power 3.1 (Heinrich Heine University, Dusseldorf, Germany), assuming a statistical power (1-β) of 80 %, significance level (a) of 5 %, and small effect size of 0.1, resulting in a total sample size requirement of 785.

### 2.2 Statistical analysis

Categorical data were reported as frequency and percentage. Pearson's chi-square test was used to analyze the categorical variables and frequencies of respondents' willingness to initiate rescue in each of the six situations (2 bystander conditions × 3 scenarios regarding the presence of competent parties). *P* < 0.05 indicated statistical significance. All statistical analyses were performed using SPSS version 27.0.

### Ethical approval

Ethical approval for this study was granted by the 10.13039/501100011722Yokohama National University Human Research Ethics Committee (#2023–13). Informed consent was obtained from all participants.

## 3 Results

Table 1 shows the respondents’ characteristics. Of the 1088 respondents, 380 (34.9 %) and 708 (65.1 %) were female and male, respectively, with the proportion of male respondents being higher than that of the Japanese population structure. A higher proportion of respondents were older—in their 40s and 50s. Overall, 714 (65.6 %) respondents had not attended an AED training course, and of the 374 (34.4 %) respondents who did, 201 (53.7 %) had attended one more than five years prior.

**Table 1:** Respondent demographicsf.

Demographic		*n*	(%)
Gender			
	Men	708	(65.1)
	Women	380	(34.9)
Age (in bands)			
	≤29	48	(4.4)
	30–39	120	(11.0)
	40–49	224	(20.6)
	50–59	298	(27.4)
	≥60	398	(36.6)
AED training experience			
	no	714	(65.6)
	yes	374	(34.4)
	Never attended		
	Attended ≥5 years	201	(53.7)
	Attended 3–5 years ago	85	(22.7)
	Attended 1–3 years ago	49	(13.1)
	Attended 6 months‒1 year ago	24	(6.4)
	Attended ≤6 months ago	15	(4.0)

Note: Abbreviations: AED: automated external defibrillator.

Percentages when N is1,088.

To grasp the bystander effects and the effects of competent parties together, we analyzed the data as shown in Table 2. This table shows that more than 955 (87.8 %) respondents in sparse situations and more than 821 (75.2 %) in crowded situations showed a willingness to initiate rescue by calling for help. No significant differences were found in the identification of competent parties in sparse and crowded situations. However, in sparse situations, the number of respondents who were willing to initiate a rescue by retrieving an AED and using an AED decreased significantly when competent parties were identified. For example, 466 (42.8 %) respondents were willing to initiate a rescue by using an AED when station staff, security guards, or kiosk staff were not identified; this number reduced to 384 (35.3 %) when kiosk staff were identified and to 353 (32.4 %) when station staff or security guards were identified.

**Table 2:** Willingness to rescue when identifying competent parties in sparse/crowded situations.

Sparse/crowded	Identification of competent parties	Rescue by running up				Rescue by retrieving an AED				Rescue by using an AED			
		*n*	(%)	*χ*^*２*^	*p*	*n*	(%)	*χ*^*２*^	*p*	*n*	(%)	*χ*^*２*^	*p*
				0.782	0.68			12.477	0.002			26.93	<0.001
Sparse	Station staff or security guards	955	(87.8)			576	(52.9)			353	(32.4)		
	Kiosk staff	967	(88.9)			625	(57.4)			384	(35.3)		
	Neither station staff and security guards nor kiosk staff	956	(87.9)			657	(60.4)			466	(42.8)		
				0.164	0.92			0.287	0.866			4.625	0.099
Crowded	Station staff or security guards	829	(76.2)			523	(46.1)			333	(30.6)		
	Kiosk staff	821	(75.2)			526	(48.3)			341	(31.3)		
	Neither station staff and security guards nor kiosk staff	824	(75.7)			535	(49.1)			377	(34.7)		

Note: Participants who answered “willing to” and “probably willing to” represent n and the percentage, respectively.

Abbreviations: AED: automated external defibrillator.

Percentages when N is 1088.

n: number of people who responded “willing to” and “probably willing to” under sparse or crowded situations in each of the three scenarios of competitive parties (presence of station staff and security guards within visual range, platform kiosk staff within visual range, and neither station staff and security guards nor platform kiosk staff within visual range).

Subsequent analyses were performed to test the hypotheses as shown in Table 3. First, in the sparse situation, 916 (84.2 %) respondents stated that they were willing to initiate a rescue by calling for help, 538 (49.4 %) were willing to initiate a rescue by retrieving an AED, and only 334 (30.7 %) were willing to initiate a rescue by using an AED. Thus, the willingness to initiate a rescue decreased across the three conditions. A similar result was obtained in the crowded situation.

**Table 3:** Willingness to rescue under the sparse/crowded situation and recognition of competent parties.

		Rescue by running up				Rescue by retrieving an AED				Rescue by using an AED			
		*n*	(%)	*χ*^*２*^	*p*	*n*	(%)	*χ*^*２*^	*p*	*n*	(%)	*χ*^*２*^	*p*
Bystander effects				58.22	<0.001			9.587	0.002			1.996	0.158
	sparse	916	(84.2)			538	(49.4)			334	(30.7)		
	crowded	767	(70.5)			466	(42.8)			304	(27.9)		
Identification of competent parties				0.667	0.716			3.782	0.151			11.75	0.003
	station staff or security guards	806	(74.1)			475	(43.7)			298	(27.4)		
	kiosk staff	809	(74.5)			506	(46.5)			304	(27.9)		
	neither station staff and security guards nor kiosk staff	811	(74.5)			519	(47.7)			364	(33.5)		

Note: Participants who answered “willing to” and “probably willing to” represent n and the percentage, respectively.

Abbreviations: AED: automated external defibrillator.

Percentages when N is 1088.

n (Bystander effects): the number of people who responded “willing to” and “probably willing to” under sparse or crowded situation in all three scenarios of competitive parties (presence of station staff and security guards were within visual range, platform kiosk staff were within visual range, and neither station staff and security guards nor platform kiosk staff were within visual range).

Identification of competent parties): the number of people who responded “willing to” and “probably willing to” when recognizing station staff or security guards, kiosk staff, or neither station staff, security guards, nor kiosk staff under sparse and crowded situations.

The proportion of respondents willing to initiate a rescue by calling for help, retrieving an AED, or using an AED was compared between the sparse and crowded situations. The willingness to rescue by calling for help and by retrieving an AED decreased with an increase in the number of people present (e.g., in the sparse situation, 916 [84.2 %] respondents were willing to initiate a rescue by calling for help [rescue by running up] compared with 767 [70.5 %] in the crowded situation). Moreover, no significant difference was observed in willingness to initiate a rescue by using an AED between sparse and crowded situations. Consequently, Hypothesis 1 was partially supported.

Next, we compared respondents’ willingness to initiate a rescue under the three behaviors when competent parties are identified, as shown in Table 3. More than 806 (74.1 %) and 475 (43.7 %) respondents were willing to initiate a rescue by calling for help and by retrieving an AED, respectively, notwithstanding their identification of competent parties. For example, regarding the rescue behavior of calling for help, no significant differences were observed between the identification of (a) station staff or security guards, (b) kiosk staff, and (c) neither station staff and security guards nor kiosk staff (806 [74.1 %], 809 [74.5 %], and 811 [74.5 %], respectively). However, for the behavior of rescuing by using an AED, significant differences were observed among the varying settings of competent parties. The percentages of respondents willing to initiate a rescue by using an AED diminished in the following order: identifying neither station staff and security guards nor kiosk staff, identifying kiosk staff, and identifying station staff or security guards; this revealed the effects of competent parties.

For example, while 364 (33.5 %) respondents were willing to initiate a rescue by using an AED when identifying neither station staff and security guards nor kiosk staff, this figure reduced to 304 (27.9 %) when they identified kiosk staff and to 298 (27.4 %) when they identified station staff or security guards. Consequently, Hypothesis 2 was partially supported.

Overall, bystander effects were detected in this study, implying that the presence of others influences the behavioral intervention of bystanders who witness an OHCA. In addition, this study showed that the perceived presence of competent parties at the location of a cardiac arrest reduced bystanders’ willingness to initiate rescue under certain circumstances.

In addition to the above findings, we explored whether bystander effects were related to actors’ demographic variables, such as gender, age, and AED training experience.

**Fig. 2:**
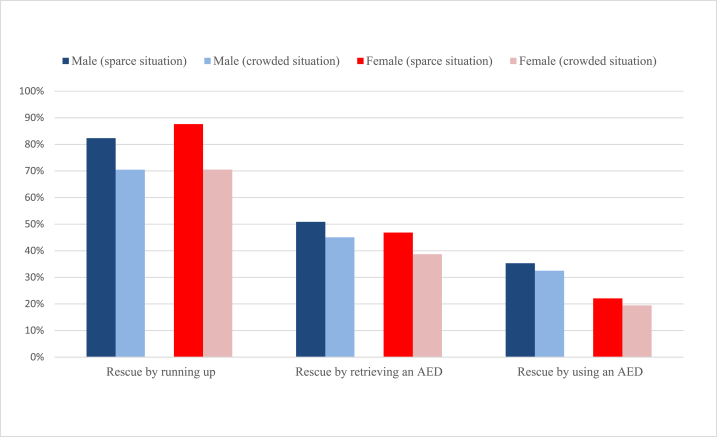
Willingness to rescue under the sparce/crowded situation by gender.

First, Fig. 2 shows the proportional changes in the willingness to rescue from the sparse to crowded situations of male and female respondents. For both males and females, there were significant decreases in the rates of respondents’ willingness to rescue by running up and by retrieving an AED from sparse to crowded situations (*p* < 0.01). However, no significant reduction was observed in the willingness to rescue by using an AED for both males and females. These results may suggest that bystander effects occur regardless of gender.

**Fig. 3:**
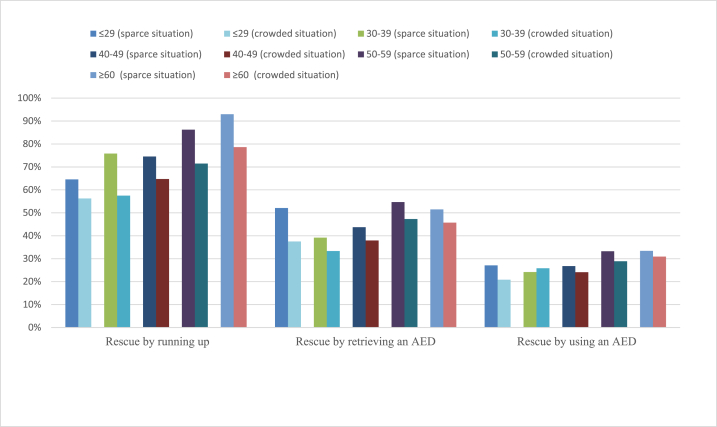
Willingness to rescue under the sparce/crowded situation by age.

Subsequently, bystander effects were explored in terms of effects of respondents’ age. As shown in Fig. 3, the rate of willingness to rescue by running up significantly decreased from sparse to crowded situations among the age groups of 30–39, 40–49, and 60 and older (*p* < 0.05), although there were no such clear differences for the age group 29 years old or younger. However, for each generation, a significant reduction was not detected in the willingness to rescue by retrieving an AED or by using an AED, except for rescue by retrieving an AED for the group 29 years old or younger.

**Fig. 4:**
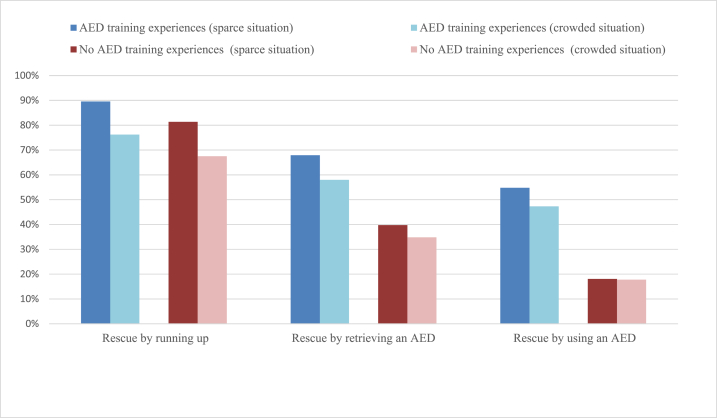
Willingness to rescue under the sparce/crowded situation by AED training experiences.

Fig. 4 shows that bystander effects occur for those who have never experienced AED training, as well as for those who have experienced it. Previous studies have shown that those who attended AED training courses gained the necessary skills, knowledge, and attitudes for rescue. However, for both parties, the willingness to rescue by running up and by retrieving an AED significantly reduced from sparse to crowded situations (*p* < 0.01). This reduction was observed in willingness to rescue by using an AED for those who attended AED training. However, for those who had not attended AED training, a reduction was not detected. Subsequently, although experiences of AED training may influence the willingness to rescue through different intervening behaviors, there is no evidence to believe that experiences of AED training affect bystander effects.

Throughout the analyses of the effects of demographic factors on bystander effects, we did not discover any concrete, uniform relationships between the demographic factors and bystander effects, although it was shown that demographic factors may be related to actors’ attitudes toward certain rescue interventions.

## 4 Discussion

This study revealed that the bystander effect proposed by social psychology is present in AED lifesaving situations. The use of social psychology knowledge to understand the bystander effect in human behavior has implications for how interventions can be designed to mobilize and support lay responders.

First, it is useful to widely inform the public, station staff, security guards, emergency medical technicians (EMTs), and others that the bystander effect may exist at the scene of a cardiac arrest. This is because understanding that bystanders’ rescue actions may differ depending on the number of strangers that are present is important for conducting lifesaving actions at the scene of a cardiac arrest, where an immediate response is required.

Second, the fact that many bystanders indicated a willingness to undertake rescue actions (e.g., calling for help) has implications for creating a life-saving system. Designing life-saving systems with the understanding that many bystanders are willing to undertake rescue actions in response to calls for help could be effective.

Third, the public's reluctance to engage in rescue actions involving AEDs suggests that a life-saving mechanism, in which competent individuals play a central role in AED-related rescue efforts, could be effective. Competent individuals are expected to assume a rescuer's role based on the understanding that the public's willingness to intervene may decrease when they are aware of the presence of competent parties. This, in turn, may imply that station staff, security guards, and kiosk staff should acknowledge that they are seen as competent parties in the eyes of the general public.

Fourth, the fact that AEDs are often located in large-scale visitor attraction facilities suggests the usefulness of developing a system that considers the possibility of the bystander effect. Japan has no government regulations regarding the location of AEDs. The Ministry of Health, Labour and Welfare has published “Guidelines for the Proper Placement of AEDs” [39], which provides guidelines on desirable locations and methods of installation. Thirteen types of facilities and locations are recommended for installation (e.g., train stations and airports, large-scale commercial facilities such as department stores and supermarkets, entertainment facilities, large-scale hotels, and convention centers). While they are undoubtedly appropriate locations for installation, the large number of people congregating there may create a bystander effect.

One strategy to address this problem is to train neighbors who frequent the installation or facility where a cardiac arrest or illness has occurred in the use of AEDs. This is because bystanders tend to intervene when familiarity with the environment is high [2], and training provides an opportunity to become more familiar with the location/area. Additionally, bystanders are more likely to intervene when networks/teams are formed among neighbors, and good teamwork/group cohesion is increased through training [8,40]. Increased CPR capacity at the community level leads to higher rates of bystander rescue and survivor discharge [41,42].

### 4.1 Limitations

Our study has some limitations. While exploring the bystander effect and individuals' intentions regarding interventions during a medical emergency and the use of CPR or an AED can contribute to the literature, the methodological limitations affect the generalizability and conclusions of our study. First, as mentioned in the results section, the sample does not represent the entire adult population of Japan. Although our survey aimed to uniformly represent the Japanese population, the samples we obtained from the data pool of one commercial research company were skewed, with a higher proportion of male respondents and older ages. Second, a variety of lifesaving behaviors, specifically calling the emergency number, performing chest compressions and ventilations, and interacting with paramedics, were not adequately examined in this study. This is because this study focused on lifesaving AED use; however, it may have been more useful to conduct an analysis that included the various behaviors described above. Third, our data were collected through self-report, which means that they were affected by common-method bias and social-desirability bias. Our study did not examine actual behavior but focused solely on people's self-reported intentions and beliefs. Fourth, our study recruited a convenience sample; thus, the participants who agreed to participate may have had certain characteristics that could confound our results.

Future studies could include interviews with bystanders who were involved in lifesaving behaviors (e.g., what were the factors that motivated or discouraged rescue actions) and interviews with EMTs who observed bystanders’ rescue behavior (e.g., what situations were observed as facilitators of bystander lifesaving). Furthermore, since Japan is known to have a large amount of research data on OHCA as well as on AEDs, as follow-up research to this study, we would like to examine whether the presence of a large number of people at the scene in an actual OHCA situation is related to whether layperson CPR is performed.

## 5 Conclusions

The purpose of this study was to identify the presence of others as a factor inhibiting rescue actions using an AED from a social psychology perspective. Social psychology research has investigated the willingness of bystanders who witness an emergency situation to initiate rescue actions, presenting the well-known concept of the bystander effect, in which the presence of others inhibits rescue actions. However, the bystander effect has not been specifically examined in the context of cardiac arrest, pointing to the need for more knowledge regarding the bystander effect in relation to rescue with AEDs.

This study presents four key conclusions. First, the bystander effect was observed for bystanders who witnessed an OHCA. Second, bystanders were less willing to rescue those in need when they identified competent parties among themselves. This tendency was observed in both crowded and sparse situations. Third, there were several levels of life-saving behavior; furthermore, there was a predominant decrease in willingness to initiate a rescue effort as the situation progressed from “rescue by running and calling for help” to “rescue by retrieving an AED” and “rescue by using an AED.” Fourth, a bystander effect was observed in “rescue by running and calling for help” and “rescue by retrieving an AED” but not in “rescue by using an AED.” Willingness to “rescue by using an AED” was low in both the sparse and crowded situations, suggesting that there are significant invisible barriers to rescue efforts involving AED use.

The current analysis results were derived from the integration of sociopsychological phenomena related to the bystander effect and those related to patient outcomes after OHCA. These new insights imply the potential benefits of utilizing social science perspectives regarding life-saving systems and CPR education.

## Ethical approval statement

The study was conducted according to the guidelines of the Declaration of Helsinki. Ethical approval for this study was granted by the Yokohama National University Human Research Ethics Committee (#2023–13).

## Funding

We gratefully acknowledge the financial support provided by JSPS KAKENHI (Grant Numbers JP20H01527 and JP24K00281) for this research.

## Data availability statement

Data will be made available on request to the corresponding author.

## CRediT authorship contribution statement

**Hideko Kono:** Writing – review & editing, Writing – original draft, Validation, Supervision, Project administration, Methodology, Investigation, Funding acquisition, Formal analysis, Data curation, Conceptualization. **Koichi Takaishi:** Writing – review & editing, Writing – original draft, Validation, Supervision, Project administration, Methodology, Investigation, Formal analysis, Data curation, Conceptualization. **Masaya Onuma:** Writing – review & editing, Validation, Supervision, Project administration, Methodology, Investigation, Formal analysis, Data curation, Conceptualization. **Michi Fukushima:** Writing – review & editing, Validation, Supervision, Project administration, Methodology, Investigation, Data curation. **Ryosuke Takeuchi:** Writing – review & editing, Validation, Supervision, Project administration, Methodology, Investigation, Data curation.

## Declaration of competing interest

The authors declare no conflict of interest.
