# Supplementary material for: Bystanders’ willingness to assist using automated external defibrillators during cardiac arrest
Source: Heliyon. 2024 Sep 2;10(17):e37316. doi: 10.1016/j.heliyon.2024.e37316 (PMC11407973; doi:10.1016/j.heliyon.2024.e37316)
Supplement: Multimedia component 1 [file mmc1.docx]

「救助、救命に対する意識」に関するアンケート

このアンケートでは、「見知らぬ人が目の前で倒れ、反応がなく呼吸が普段通りではない状態であった」時の救助、救命に関する意識を伺います。「正しい内容」「間違った内容」といったものはありません。お考えをそのままお答えください。

研究成果は、救助・救命比率を高める仕組みの検討に生かしたいと考えており、ご協力をお願い申し上げます。

＜用語説明＞AED

AED（自動体外式除細動器）は、心停止（症状例：反応がなく呼吸が普段通りではなくなる）を起こした際、電気ショックで心臓の状態を正常に戻す小型器械です。電源を入れると音声で使い方が指示されるので、誰もが使用し救命活動を行うことができます。

多くの駅、学校、公園に設置されており、マラソン、お祭りなどのイベント時にも設置されます。

アンケート調査へのご協力のお願い

このアンケート調査は、救助、救命に関する意識をお尋ねするものです。科学研究費の助成を受けて実施するものであり、研究成果は、救助・救命比率を高める仕組みの検討に生かしたいと考えております。ご協力をお願い申し上げます。

＜調査実施責任者＞

横浜国立大学大学院国際社会科学研究院　河野英子（教授）

＜基本的な考え方＞

アンケートの目的は、個人の特徴を分析することではなく、回答者の全体的な傾向を検討することにあります。そのため、特定の個人のデータを抜き出して分析するといったことはございません。「正しい内容」「間違った内容」といったものはありません。お考えをそのままお答えください。

研究へのご協力は任意であり、参加しないことで不利益はありません。同意をいつでも撤回することが出来ます。

＜個人情報の保護＞

アンケート調査は無記名で行われます。回答者氏名などの個人情報は収集いたしません。研究終了後には破棄致します。

＜回答結果の公表＞

ご回答いただいた内容は、本研究の分析にのみ使用いたします。分析結果は、研究の成果として学会発表や学術雑誌等で公表致します。

同意する　　□回答をやめる　□回答する

**Q1.** シーン１

あなたが一人で駅のホームにいた時、見知らぬ人が目の前で倒れたと想像してください。反応がなく呼吸が普段通りではないようでした。平日午後の時間帯で、ホームにいる人はまばらでした。

状況a

駅員や警備員があなたの見える範囲にいるとき、あなたはどのように行動すると思いますか？（単一解答）

|  | しない | おそらくしない | どちらともいえない | おそらくする | する |
| --- | --- | --- | --- | --- | --- |
| 駆け寄る、助けを呼ぶなど、救助のために行動する | □ | □ | □ | □ | □ |
| AEDを取りに行くなど、救命のために行動する | □ | □ | □ | □ | □ |
| AEDを使用した救命行為をする | □ | □ | □ | □ | □ |

**Q2.** シーン１

あなたが一人で駅のホームにいた時、見知らぬ人が目の前で倒れたと想像してください。反応がなく呼吸が普段通りではないようでした。平日午後の時間帯で、ホームにいる人はまばらでした。

状況b

ホーム売店の店員があなたの見える範囲にいるとき、あなたはどのように行動すると思いますか？（単一解答）

|  | しない | おそらくしない | どちらともいえない | おそらくする | する |
| --- | --- | --- | --- | --- | --- |
| 駆け寄る、助けを呼ぶなど、救助のために行動する | □ | □ | □ | □ | □ |
| AEDを取りに行くなど、救命のために行動する | □ | □ | □ | □ | □ |
| AEDを使用した救命行為をする | □ | □ | □ | □ | □ |

**Q3.** シーン１

あなたが一人で駅のホームにいた時、見知らぬ人が目の前で倒れたと想像してください。反応がなく呼吸が普段通りではないようでした。平日午後の時間帯で、ホームにいる人はまばらでした。

状況ｃ

駅員も警備員もホームの売店の店員もあなたの見える範囲にいないとき、あなたはどのように行動すると思いますか？（単一解答）

|  | しない | おそらくしない | どちらともいえない | おそらくする | する |
| --- | --- | --- | --- | --- | --- |
| 駆け寄る、助けを呼ぶなど、救助のために行動する | □ | □ | □ | □ | □ |
| AEDを取りに行くなど、救命のために行動する | □ | □ | □ | □ | □ |
| AEDを使用した救命行為をする | □ | □ | □ | □ | □ |

**Q4.** シーン２

あなたが一人で駅のホームに降りた時、見知らぬ人が目の前で倒れたと想像してください。反応がなく呼吸が普段通りではないようでした。ラッシュの時間帯で、ホームは多くの乗降客で混雑していました。

状況d

駅員や警備員があなたの見える範囲にいるとき、あなたはどのように行動すると思いますか？（単一解答）

|  | しない | おそらくしない | どちらともいえない | おそらくする | する |
| --- | --- | --- | --- | --- | --- |
| 駆け寄る、助けを呼ぶなど、救助のために行動する | □ | □ | □ | □ | □ |
| AEDを取りに行くなど、救命のために行動する | □ | □ | □ | □ | □ |
| AEDを使用した救命行為をする | □ | □ | □ | □ | □ |

**Q5.** シーン２

あなたが一人で駅のホームに降りた時、見知らぬ人が目の前で倒れたと想像してください。反応がなく呼吸が普段通りではないようでした。ラッシュの時間帯で、ホームは多くの乗降客で混雑していました。

状況e

ホーム売店の店員があなたの見える範囲にいるとき、あなたはどのように行動すると思いますか？（単一解答）

|  | しない | おそらくしない | どちらともいえない | おそらくする | する |
| --- | --- | --- | --- | --- | --- |
| 駆け寄る、助けを呼ぶなど、救助のために行動する | □ | □ | □ | □ | □ |
| AEDを取りに行くなど、救命のために行動する | □ | □ | □ | □ | □ |
| AEDを使用した救命行為をする | □ | □ | □ | □ | □ |

**Q6.** シーン２

あなたが一人で駅のホームに降りた時、見知らぬ人が目の前で倒れたと想像してください。反応がなく呼吸が普段通りではないようでした。ラッシュの時間帯で、ホームは多くの乗降客で混雑していました。

状況f

駅員も警備員もホームの売店の店員もあなたの見える範囲にいないとき、あなたはどのように行動すると思いますか？（単一解答）

|  | しない | おそらくしない | どちらともいえない | おそらくする | する |
| --- | --- | --- | --- | --- | --- |
| 駆け寄る、助けを呼ぶなど、救助のために行動する | □ | □ | □ | □ | □ |
| AEDを取りに行くなど、救命のために行動する | □ | □ | □ | □ | □ |
| AEDを使用した救命行為をする | □ | □ | □ | □ | □ |

＜人口統計変数＞

AEDを使用する訓練を受講したことがありますか？（単一解答）

　□ある

　□ない

最後に訓練を受講したのは、おおむねいつ頃ですか？（単一解答）

　□受講したことはない

　□5年以上前

　□3年以上5年未満前

　□1年以上3年未満前

　□6か月以上1年未満前

　□6か月未満

あなたは医療関係者（医師、看護師、救命救急士等）ですか。（単一解答）

　□はい

　□いいえ

あなたは駅員または警備員ですか。（単一解答）

　□はい

　□いいえ

性別（単一解答）

　□男性

　□女性

　□回答しない

年齢（単一解答）

　□29歳未満

□30～39歳

□40～49歳

□50～59歳

□60歳以上

ご協力ありがとうございました。
