# Supplementary material for: Bystanders’ willingness to assist using automated external defibrillators during cardiac arrest
Source: Heliyon. 2024 Sep 2;10(17):e37316. doi: 10.1016/j.heliyon.2024.e37316 (PMC11407973; doi:10.1016/j.heliyon.2024.e37316)
Supplement: Multimedia component 2 [file mmc2.docx]

（English translation）**Questionnaire on Willingness to Rescue and Save Lives**

This questionnaire asks about your willingness of rescue and lifesaving measures when a stranger collapses in front of you, unresponsive and not breathing normally. There are no "right" or "wrong" answers. Please answer the question as it is in your opinion.

We would like to utilize the results of this research in the study of a system to improve the ratio of rescue and lifesaving, and would appreciate your cooperation.

<Explanation of Terms> AED

AED (Automated External Defibrillator) is a small device that restores the heart to normal condition with an electric shock in the case of cardiac arrest (symptom: no response and breathing is not normal). When turned on, the device provides voice instructions on how to use it, allowing anyone to use it and perform lifesaving activities.

The device is installed at many train stations, schools, and parks, and is also set up during events such as marathons and festivals.

<Request for your cooperation in the survey>

This survey is intended to inquire about your willingness of rescue and lifesaving activities. The survey is supported by a grant from the Japan Society for the Promotion of Science (JSPS), and we would like to utilize the results of the survey in the development of a system to increase the ratio of rescues and lifesaving. We would appreciate your cooperation.

<Person Responsible for Conducting the Survey＞

Professor Hideko Kono, International Graduate School of Social Sciences, Yokohama National University

<Basic Concept＞

The purpose of the survey is not to analyze individual characteristics, but to examine overall trends among respondents. Therefore, we do not extract data from specific individuals for analysis. There are no "right" or "wrong" answers. Please answer the questions as you see fit.

Your participation in the study is voluntary, and you will not be disadvantaged by not participating. You can withdraw your consent at any time.

＜Protection of Personal Information＞

The survey will be conducted anonymously. Personal information such as respondents' names will not be collected. We will discard such information after the completion of the study.

<Disclosure of response results＞

We will use your answers only for the analysis of this research. The results of the analysis will be published as the outcome of the research at academic conferences and in academic journals.

I agree.　　□I will stop answering.　　□I will answer.

**Q1. scene 1**

**Imagine that you were alone on a station platform when a stranger collapsed in front of you. He was unresponsive and his breathing did not seem to be normal.** **It was a weekday afternoon, and there were few people on the platform.**

**Situation a**

**How do you think you would act if station staff or security guards were within your visual** **range? （SA）**

|  | Not willing to | Probably not willing to | Neither willing nor not willing to | Probably not willing to | Willing to |
| --- | --- | --- | --- | --- | --- |
| Rescue by running and calling for help | □ | □ | □ | □ | □ |
| Rescue by retrieving an AED | □ | □ | □ | □ | □ |
| Rescue by using an AED | □ | □ | □ | □ | □ |

**Q2. scene 1**

**Imagine that you were alone on a station platform when a stranger collapsed in front of you. He was unresponsive and his breathing did not seem to be normal. It was a weekday afternoon, and there were few people on the platform.**

**Situation b**

**How do you think you would behave if the platform kiosk staff were within visual range ? （SA）**

|  | Not willing to | Probably not willing to | Neither willing nor not willing to | Probably not willing to | Willing to |
| --- | --- | --- | --- | --- | --- |
| Rescue by running and calling for help | □ | □ | □ | □ | □ |
| Rescue by retrieving an AED | □ | □ | □ | □ | □ |
| Rescue by using an AED | □ | □ | □ | □ | □ |

**Q3. scene 1**

**Imagine that you are alone on a station platform and a stranger collapsed in front of you. He was unresponsive and his breathing did not seem to be normal. It was a weekday afternoon, and there were few people on the platform.**

**Situation c**

**How do you think you would act if neither station staff and security guards nor platform kiosk staff were within visual rang? （SA）**

|  | Not willing to | Probably not willing to | Neither willing nor not willing to | Probably not willing to | Willing to |
| --- | --- | --- | --- | --- | --- |
| Rescue by running and calling for help | □ | □ | □ | □ | □ |
| Rescue by retrieving an AED | □ | □ | □ | □ | □ |
| Rescue by using an AED | □ | □ | □ | □ | □ |

**Q4. scene 2**

**Imagine that you are alone on a station platform and a stranger collapsed in front of you. He was unresponsive and his breathing did not seem to be normal. It was rush hour and the platform was crowded with passengers.**

**Situation d**

**How do you think you would act if station staff or security guards were within your visual range? （SA）**

|  | Not willing to | Probably not willing to | Neither willing nor not willing to | Probably not willing to | Willing to |
| --- | --- | --- | --- | --- | --- |
| Rescue by running and calling for help | □ | □ | □ | □ | □ |
| Rescue by retrieving an AED | □ | □ | □ | □ | □ |
| Rescue by using an AED | □ | □ | □ | □ | □ |

**Q5. scene 2**

**Imagine that you are alone on a station platform and a stranger collapsed in front of you. He was unresponsive and his breathing did not seem to be normal. It was rush hour and the platform was crowded with passengers.**

**Situation e**

**How do you think you would act when the platform kiosk staff were within visual range? （SA）**

|  | Not willing to | Probably not willing to | Neither willing nor not willing to | Probably not willing to | Willing to |
| --- | --- | --- | --- | --- | --- |
| Rescue by running and calling for help | □ | □ | □ | □ | □ |
| Rescue by retrieving an AED | □ | □ | □ | □ | □ |
| Rescue by using an AED | □ | □ | □ | □ | □ |

**Q6. scene 2**

**Imagine that you are alone on a station platform and a stranger collapses in front of you. He was unresponsive and his breathing did not seem to be normal. It was rush hour and the platform was crowded with passengers.**

**Situation f**

**How do you think you would act if neither station staff and security guards nor platform kiosk staff were within visual range? （SA）**

|  | Not willing to | Probably not willing to | Neither willing nor not willing to | Probably not willing to | Willing to |
| --- | --- | --- | --- | --- | --- |
| Rescue by running and calling for help | □ | □ | □ | □ | □ |
| Rescue by retrieving an AED | □ | □ | □ | □ | □ |
| Rescue by using an AED | □ | □ | □ | □ | □ |

<Demographic variables>

**Have you ever attended training to use an AED? （SA）**

　□Yes

　□No

**When was the last time you attended training? （SA）**

□never attended

□attended more than 5 yeads ago

□attended 3 to 5 years ago

□attended 1 to 3 years ago

□attended 6 months to 1 year ago

□attended less than 6 months ago

**Are you a medical professional (doctor, nurse, paramedic, etc.)? （SA）**

□Yes

□No

**Are you a station staff or security guard? （SA）**

□Yes

□No

**Gender（SA）**

□Male

□Female

□Do not answer

**Age（SA）**

□younger than 29 years

□30～39

□40～49

□50～59

□60 and older

Thank you for your cooperation.
